# The combined effect of obesity and aging on human sperm DNA methylation signatures: inclusion of BMI in the paternal germ line age prediction model

**DOI:** 10.1038/s41598-020-71979-8

**Published:** 2020-09-21

**Authors:** Albert Salas-Huetos, Emma R. James, Dallin S. Broberg, Kenneth I. Aston, Douglas T. Carrell, Timothy G. Jenkins

**Affiliations:** 1 Andrology and IVF Laboratory, Division of Urology, Department of Surgery, University of Utah School of Medicine, Salt Lake City, UT 84108 USA; 2 Department of Human Genetics, University of Utah School of Medicine, Salt Lake City, UT 84108 USA; 3 Department of Physiology and Developmental Biology, Brigham Young University, Provo, UT 84062 USA

**Keywords:** Genetics research, Ageing, Urology

## Abstract

Male aging and obesity have both been shown to contribute to declines in fertility in men. Recent work in aging has shown consistent epigenetic changes to sperm as a man ages. In fact, our lab has built a tool that utilizes DNA methylation signatures from sperm to effectively predict an individual’s age. Herein, we performed this preliminary cohort study to determine if increased BMI accelerates the epigenetic aging in sperm. A total of 96 participants were divided into four age groups (22–24, 30, 40–41, and > 48 years of age) and additionally parsed into two BMI sub-categories (normal and high/obese). We found no statistically significant epigenetic age acceleration. However, it is important to note that within each age category, high BMI individuals were predicted to be older on average than their actual age (~ 1.4 years), which was not observed in the normal BMI group. To further investigate this, we re-trained a model using only the present data with and without BMI as a feature. We found a modest but non-significant improvement in prediction with BMI [r^2^ = 0.8814, mean absolute error (MAE) = 3.2913] compared to prediction without BMI (r^2^ = 0.8739, MAE = 3.3567). Future studies with higher numbers of age-matched individuals are needed to definitively understand the impact of BMI on epigenetic aging in sperm.

## Introduction

In developed-industrialized countries, societal factors (e.g. marriage at older ages, the pursuit of career goals and economic pressures) are determinant factors whereby couples are waiting for longer periods of time prior to having children^1^. Over the past 40 years this has resulted in a significant increase in the average age of paternity, with the mean paternal age having increased by almost 4 years^2^. These trends have created a need for research regarding factors that may affect fertility and offspring health for aged couples with assessment of contributing factors in both men and women.

Although the effect of aging on the female reproductive system is more evident (decreasing drastically at 35–40 years and ultimately resulting in an absolute barrier to achieving a pregnancy), the effects of advanced paternal age also has consequences, though they are far more subtle (e.g. in males, some authors suggest that sperm motility, among other parameters, decreases continuously between 22 and 80 years of age). The issue of advanced paternal age has begun to receive more attention in recent years because of the possible effects of aging on sperm epigenetics and therefore on fertility, pregnancy outcomes, and even offspring health^3^. In fact, some studies linked older fathers to an increased prevalence of several neuropsychiatric disorders (autism, bipolar disorder, and schizophrenia) in the offspring and others have identified potential associations to significant alterations in sperm DNA methylation patterns^4,5^.

Another recent change in developed societies are dietary modifications. Nowadays, societies are more likely to consume Western-style diets rich in processed foods, high in saturated fats, red and processed meat, and rich in sugar drinks and fried foods. This diet is well known to be associated with ailments including an increase of body weight which has been causally associated with the development of cardiovascular disease, diabetes, and some types of cancers^6^.

Over the past few decades the proportion of adult males with a body mass index (BMI) over 25 increased from 28.8% in 1980 to 36.9% in 2013^7^, indicating a considerable increase in overweight/obesity prevalence over a short period of time. Recent studies also support that male overweight/obesity status can affect sperm DNA methylation^8^ and therefore could affect fertility, pregnancy outcomes, and offspring health, as aforementioned with male aging.

Taking into account that both factors, male aging^9^ and obesity^10^, are associated with declining semen quality^11^, that both affect sperm DNA methylation patterns, and that methylation patterns can be used as a predictive function for calculating the germ-line age of sperm, as we demonstrated previously^12^, we hypothesized that BMI could potentially negatively impact epigenetic aging in the sperm. Subsequently, the potential for age acceleration in the sperm epigenome may be indicative of poor reproductive phenotypes and outcomes. Such a hypothesis is supported by data in somatic tissues where epigenetic age increased in the livers of individuals with high BMI^13^.

Therefore, the main objective of the present preliminary study was to explore the relationship between BMI and epigenetic aging in sperm. We additionally utilized the unique dataset that we compiled to compare the sperm DNA methylation patterns of obese patients and normal weight individuals to define the patterns of sperm epigenetic alterations due to increased BMI in our cohort of men.

## Results

**Figure 1:**
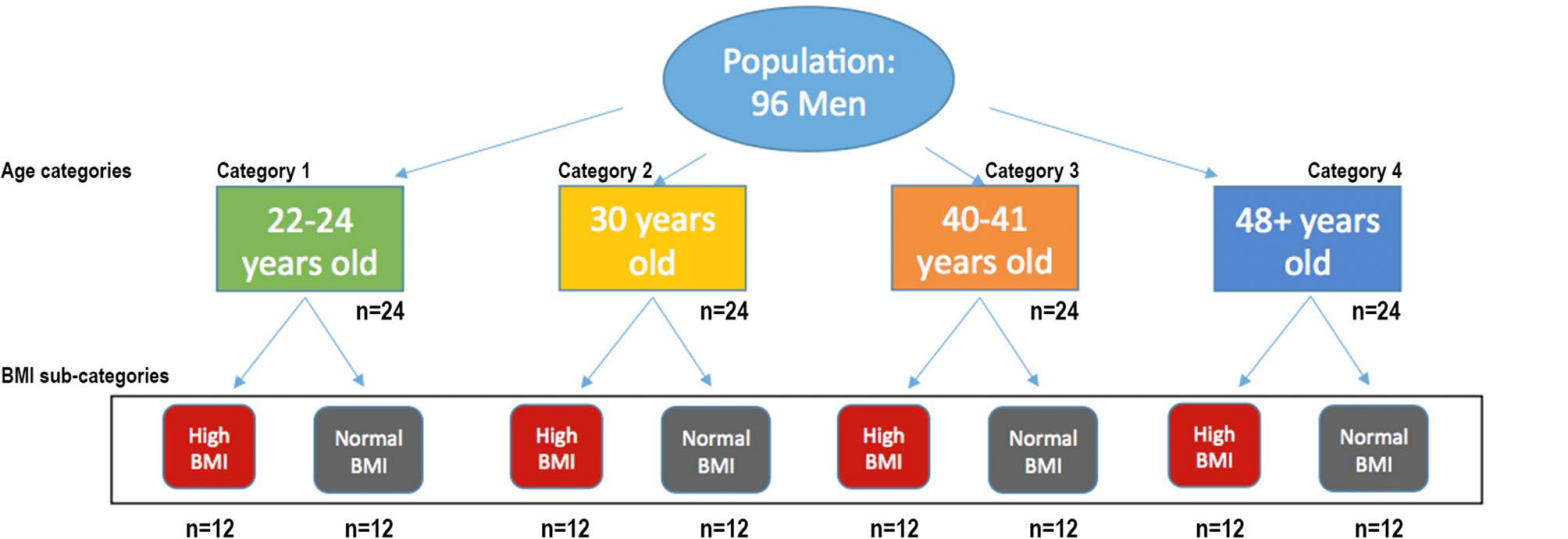
Schematic study diagram showing how participants were divided into four age categories (category 1–4) with two subset groups of BMIs (sub-categories normal and high or obese).

A total of 96 participants were included in the study and divided equally (n = 24 per category) into four different age groups or categories (22–24 years of age [category 1]; 30 years of age [category 2]; 40–41 years of age [category 3]; and > 48 years of age [category 4]). We designed the study groups in this way to ensure that sufficient representation of multiple age categories was available, and that the ages of the individuals in these categories were highly similar, making comparisons between the groups easier to achieve. These participants were subsequently divided equally (n = 12 per sub-category) into two BMI sub-categories (normal, 18.5–24.9; and high/obese, > 30.0) (Fig. 1). The general anthropometric and semen characteristics of the study participants are shown in Table 1 and were not significantly different between the high and normal BMI groups with the exception of BMI.

**Table 1:** Demographic and seminogram data of the study population.

	22–25 years of age		30 years of age		40 years of age		> 48 years of age	
	High/obese (n = 12)	Normal (n = 12)	High/obese (n = 12)	Normal (n = 12)	High/obese (n = 12)	Normal (n = 12)	High/obese (n = 12)	Normal (n = 12)
Age (years)	23.6 ± 0.3	23.5 ± 0.3	30.5 ± 0.1	30.3 ± 0.1	40.5 ± 0.4	40.8 ± 0.1	51.6 ± 0.8	56.7 ± 2.1
p-value	NS		NS		NS		NS	
BMI (kg/m^2^)	36.7 ± 2.3	20.4 ± 0.4	34.2 ± 0.9	21.9 ± 0.5	37.6 ± 2.0	23.2 ± 0.3	33.3 ± 1.0	22.9 ± 0.5
p-value	< 0.0001		< 0.0001		< 0.0001		< 0.0001	
Progressive motility (%)	44.9 ± 5.0	38.0 ± 3.7	56.6 ± 5.3	45.9 ± 3.8	37.9 ± 6.3	54.6 ± 2.8	41.8 ± 4.0	46.0 ± 3.0
p-value	NS		NS		NS		NS	
Concentration (%)	74.7 ± 14.2	71.6 ± 24.4	81.9 ± 18.7	87.1 ± 24.4	61.5 ± 19.8	64.4 ± 16.3	56.0 ± 13.2	87.7 ± 17.7
p-value	NS		NS		NS		NS	
Viability (%)	53.9 ± 3.4	49.2 ± 4.8	67.2 ± 2.0	51.7 ± 5.5	53.3 ± 3.1	51.6 ± 2.3	54.5 ± 4.1	42.1 ± 4.9
p-value	NS		NS		NS		NS	

Values are means ± standard error (SE).

*NS* not significant.

The *DLK1* locus, which we have identified as a reliable discriminator between somatic DNA and sperm DNA, contains 14 different CpG points, and is highly methylated in somatic cells and essentially unmethylated in sperm cells^14^. Following our somatic cell removal techniques, analysis of the *DLK1* locus confirmed the effectiveness of our protocols in these samples and further established the absence of contaminating somatic signals in our data set (data not shown).

Following data acquisition using the Infinium MethylationEPIC BeadChip (Illumina) and quality control measures, we analyzed intraindividual DNA methylation data for differences in methylation using several different approaches between the high BMI and normal BMI subgroups within each age category. We performed differential methylation analysis at three distinct levels: point data analysis (we assess differences in methylation at each individual CpG tiled on the array), regional analysis (where data are averaged by regions including promoters, CpG islands, and other genomic features), and global methylation (where all methylation signals are averaged across the entire array). We found no significant differences following strict multiple comparison correction (Bonferroni) in methylation between the normal and high BMI categories in our patient cohort.

**Figure 2:**
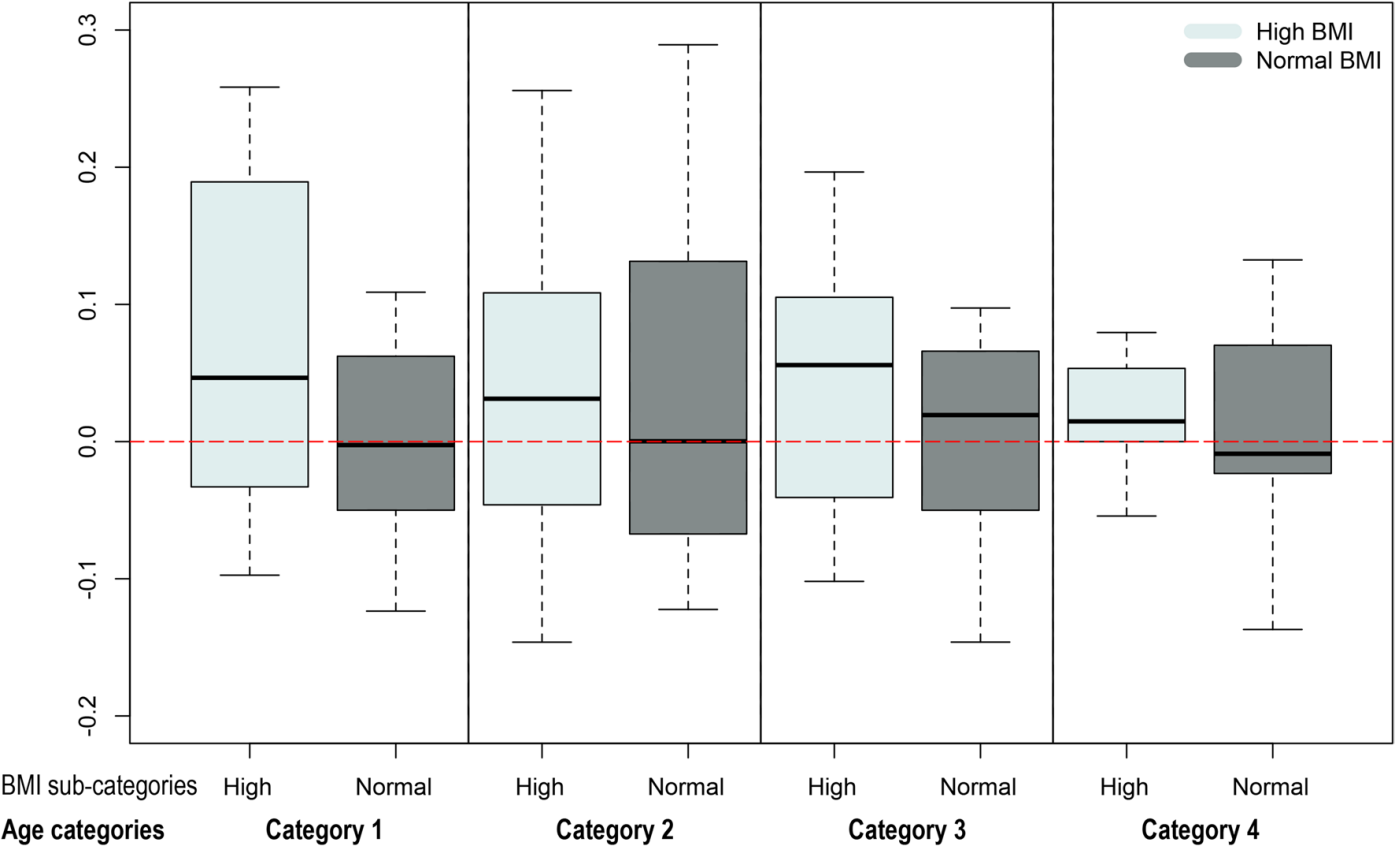
Boxplots representing the germ line age differential for each sample in their respective categories.

We applied the previously published paternal germ line aging analysis (original prediction model^12^) on the samples to determine if having a high BMI was associated with alterations in epigenetic age similar to those that have been seen in somatic tissue. Once the predicted age is identified by the model, we determine a germline age differential (GLAD) measure. This is calculated by the following equation [GLAD = (predicted age /actual age) − 1] where a number greater than zero indicates that an individual’s epigenetic age is higher than their actual age. GLAD measures were then compared across groups, and in our cohort, we found no statistical differences in GLAD between BMI subcategories. However, in each aging category, the high BMI individuals were predicted to be older (on average, ~ 1–4%) than their actual age when compared to individuals with a normal BMI. This increase in predicted age was consistent for each age category but was highest in the youngest age (22–24 years old) category (~ 4% increase in predicted age). Figure 2 depicts the germ line age differential for each sample in their respective categories.

**Figure 3:**
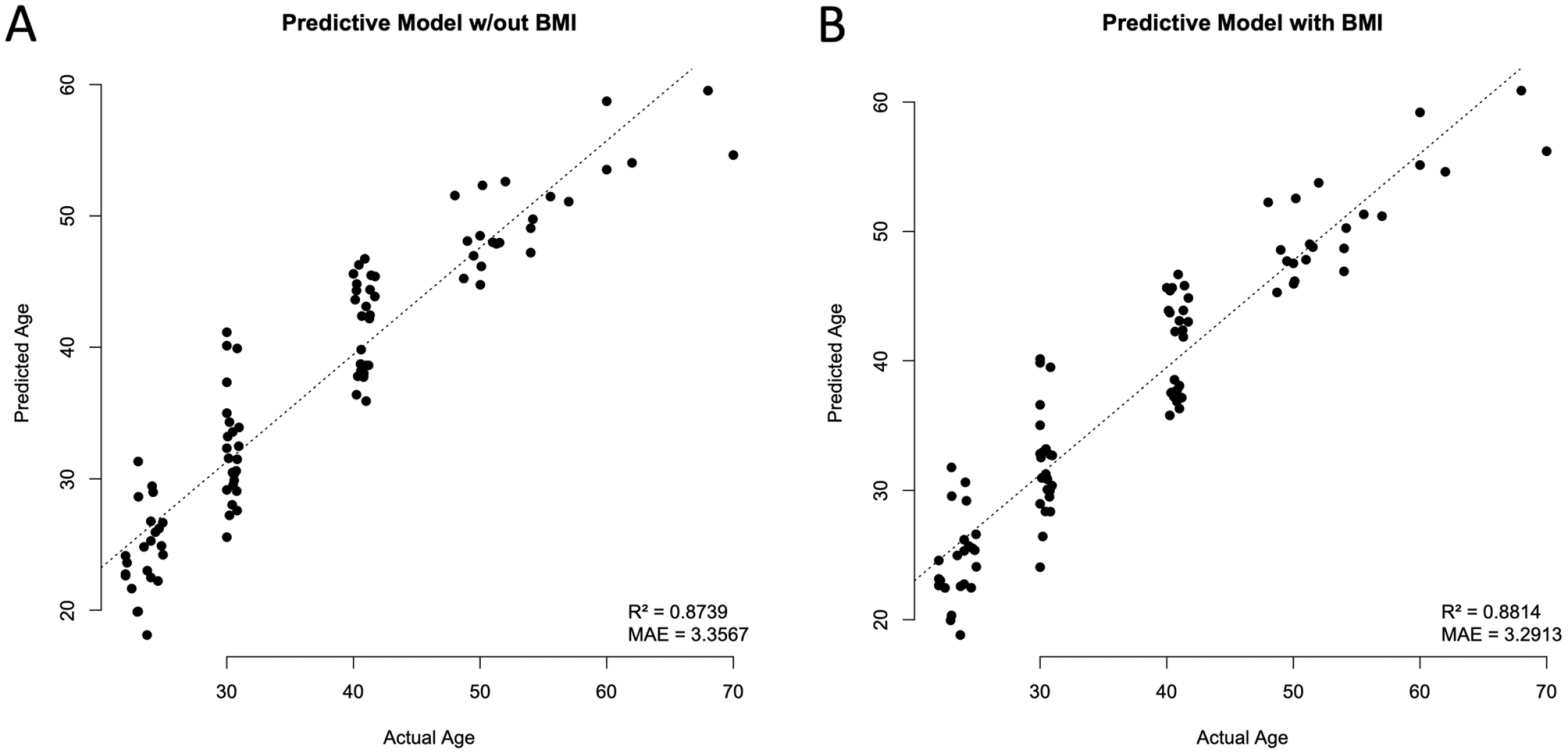
Scatter plots showing age prediction with newly constructed models of aging in the 96-sample data set (**A**) Using only the features used in the original model of aging (without BMI) and, (**B**) using the features used in the original model of aging including BMI.

Because we identified the subtle, non-significant trend toward increased epigenetic age, we sought to determine if such a subtle potential association may aid in improving the predictive capacity of the currently published calculator. To investigate this we re-trained new models using all of the same features as have been previously published^12^ with and without including BMI as a feature to determine if its inclusion could improve predictive power. Similar to our assessment of GLAD, we found a very subtle, but non-significant, improvement in predictive capacity when including BMI as a feature (Fig. 3). Specifically, the results showed that excluding BMI in the model resulted in an r^2^ of 0.8739, and a mean absolute error (MAE) of 3.3567. In the predictive model including BMI as a feature we saw an r^2^ of 0.8814 and a MAE of 3.2913.

## Discussion

Herein we report two main outcomes from our study. First, in our unique and highly selected cohort of men, we identified no significant sperm DNA methylation alterations associated with BMI. Second, we report that patterns of sperm DNA methylation aging have no statistically significant relationship with BMI in our cohort.

Our observation of a lack of a significance impact on sperm DNA methylation patterns between BMI categories contradicts previously published reports that suggest significant sperm DNA methylation differences related to obesity^15–17^. Our cohort of patients is very unique and was selected specifically for the assessment of epigenetic age differences. Because of how highly parsed our data set is, it does not represent the ideal cohort to assess differential methylation alone thus our results should be taken with caution. This is because each assessment of differential methylation was performed on limited numbers of samples and thus insignificant results are not entirely surprising. However, we felt it important to include a differential methylation screen in this assessment for completeness of our analysis.

With respect to our age acceleration analysis, despite the negative data, we feel it is important to not entirely disregard the possibility that a relationship may exist between BMI state and epigenetic age acceleration in sperm. While our data were not significant, the consistency of the trend seen was intriguing. Specifically, we found that epigenetic age acceleration was pronounced in patients with a high BMI compared to patients with a normal BMI within the same age category. In its current form, utilizing commonly available but relatively variable array data, the sperm age calculator’s output can also have some variance. Taken together with the fact that we were using relatively small sample sizes to ensure that we were able cover more of the potentially impacted age categories, the lack of significance needs to be addressed with future studies to confirm whether or not a relationship actually exists between BMI and epigenetic age in sperm. Because of the nature of the trend that we identified, it is not unreasonable to think that with more well powered studies a significant association may indeed be identified. However, based on this initial assessment, if an association does exist it is likely to be quite subtle. While typically such subtlety is troubling in an assessment of actual biological impact, in this case we expect a subtle signal because the impacts of aging on fertility are likewise quite subtle.

The principal strength of the present study is the originality of the work, because this is the first study exploring the combined effect of obesity on epigenetic aging in human sperm DNA methylation signatures. The use of the paternal germ line age prediction model allowed us to explore this relationship. The main limitation of the study is our relatively small sample size that is present in each category, because we acknowledge that we were not able to rule out the existence of small DNA methylation changes between BMI categories that could become statistically significant in larger sample sizes. Future well-designed, prospective studies on the current topic are therefore recommended that focus only on younger age groups as these appeared, from our data, to be the most likely to be impacted. Although in our preliminary cohort study we have excluded several potential confounding factors (e.g. variable semen parameters, exclusion of men who smoke), these results should be interpreted with caution because we cannot discount other possible confounding factors. Another limitation it that this study focuses on men attending a fertility clinic and therefore, the results cannot be extrapolated to other populations (e.g. general population, fertile men, etc.).

## Materials and methods

### Study design and population selected

The preliminary population-based cohort study was conducted in men attending a fertility clinic (Andrology and IVF Laboratories, University of Utah, USA) between 2008 and 2017. All experimental protocols and procedures were in accord with the Declaration of Helsinki for Medical Research involving Human Subjects and were approved by University of Utah’s Institutional Review Board (IRB). Moreover, all participants provided written informed consent approved by the University of Utah’s IRB.

The study participants were healthy, non-smoking men (22–70 years old) with a sperm concentration of at least 10 million sperm/ml. Exclusion criteria included previous vasectomy, chemotherapeutic exposure, smoking, or extremely high somatic cell content in semen analysis. The participants included in the current study (n = 96) were divided into four different age groups or categories (22–24 years of age [category 1]; 30 years of age [category 2]; 40–41 years of age [category 3]; and > 48 years of age [category 4]), which were each divided into two BMI sub-categories (normal and high/obese) to enable us to detect the combined impact of BMI and age. Participant ages were restricted to the smallest possible range within each category in order to minimize variability within categories and maximize distance between them. This design was intended to allow us to discern differences between categories even if ages were mis-predicted by 1–2 years, which would have been difficult if using larger age ranges and/or smaller distances between categories. The BMI categorization was performed using the latest World Health Organization BMI values^18^: 18.5–24.9 kg/m^2^ (normal BMI), > 30.0 kg/m^2^ (high/obese BMI).

### Sperm analysis, sperm purification and DNA isolation

Semen samples were collected by masturbation after 2–5 days of sexual abstinence. Semen analysis was assessed in fresh semen samples according the 2010 World Health Organization’s criteria^19^. After the semen analysis, semen samples were frozen following a well-stablished slow freeze protocol^20^. Briefly, semen samples were mixed in a 1:1 ratio with test yolk buffer (Irvine Scientific, CA, USA) and placed in liquid nitrogen vapors and finally maintained in liquid nitrogen until further analysis.

After thawing the sperm samples a sperm purification protocol that included stringent somatic cell lysis was performed as described previously^21^. This purification was performed by incubating sperm samples with 0.1% SDS and 0.5% Triton X-100 (in Milli-Q^®^ water), on ice followed by two high volume wash steps and a final optical microscopic examination to verify the somatic cell elimination. All samples were also epigenetically screened (*DLK1* locus) to ensure no somatic cell contamination according to a previously published method of our group^14^.

Total sperm DNA was isolated using a sperm-specific modification to the Qiagen DNeasy (QIAGEN, CA, USA) manufacturer protocol^5^, and DNA concentration and purity were determined using a Nanodrop-1000 spectrophotometer (Thermo Fisher Scientific, MA, USA).

### Bisulfite conversion and microarray analysis

Extracted sperm DNA (500 ng) was bisulfite converted with the EZ DNA Methylation kit (Zymo Research, CA, USA) according to manufacturer’s recommendations with a modification recommended for downstream utilization of Illumina array platforms. The bisulfite-converted DNA was then hybridized to Infinium^®^ MethylationEPIC microarrays (Illumina, CA, USA) and analyzed according to Illumina protocols at the University of Utah Genomics Core Facility.

### Data processing

The *minfi* Bioconductor package (*minfi*; package at https://www.bioconductor.org)^22,23^ an additional package for the R statistical computing environment v.3.5.0 (www.r-project.org)^24^ was used to process array data and generate β-values. Array data were evaluated for standard data quality indicators and subjected to SWAN normalization^25^. Normalized β-values were then logit transformed to generate M-values for further analyses.

The resulting β-values and M-values were assessed for different analyses including global methylation, CpG points and regional analysis as has been previously performed in our group^5,14,26^.

### Paternal germ line aging analysis (prediction models)

We assessed paternal ‘germ line age’ analysis with a recently constructed algorithm from our laboratory used to predict an individual’s age using sperm DNA methylation signatures^12^. Based on 51 regions of the genome we conducted an age calculation prediction for all samples. To further explore the relationship of BMI and epigenetic aging patterns we additionally trained (using the *glmnet* package in R^27^) two new models using only this dataset, one including all 51 regions identified previously, and the other using these as well as BMI as a feature. To compare the accuracy and predictive power of these models we performed linear regression for each (actual age vs. predicted age) and generated r^2^ values. To highlight the power of prediction of the models, the mean absolute error (MAE) between the actual age vs. predicted age in both models and the r^2^ values were considered and compared via two-tailed t-test. P-values of < 0.05 were considered significant.
